# Prognostic Relevance of Preoperative Immune, Inflammatory, and Nutritional Biomarkers in Patients Undergoing Gastrectomy for Resectable Gastric Adenocarcinoma: An Observational Multicentre Study

**DOI:** 10.3390/cancers16122188

**Published:** 2024-06-11

**Authors:** Jaume Tur-Martínez, Joaquín Rodríguez-Santiago, Javier Osorio, Mònica Miró, Concepción Yarnoz, Mariona Jofra, Georgina Ferret, Helena Salvador-Roses, Sonia Fernández-Ananín, Arantxa Clavell, Alexis Luna, Aurora Aldeano, Carles Olona, Judith Hermoso, Mercè Güell-Farré, Mariagiulia Dal Cero, Marta Gimeno, Natàlia Pallarès, Manuel Pera

**Affiliations:** 1 Department of Surgery, Universitat Autònoma de Barcelona, 08035 Barcelona, Spain; 2 Department of Surgery, Complex Hospitalari Universitari Moisès Broggi, 08970 Sant Joan Despí, Spain; 3 Department of Surgery, University Hospital Mútua Terrassa, 08221 Terrassa, Spain; 4 Department of Surgery, Hospital Clínic de Barcelona, 08036 Barcelona, Spain; 5 Department of Surgery, Hospital Universitari de Bellvitge, L’Hospitalet de Llobregat, 08907 Barcelona, Spain; 6 Department of Surgery, Hospital Universitario de Navarra, Universidad Pública de Navarra, 31008 Pamplona, Spain; 7 Department of Surgery, Hospital Universitari Vall d’Hebron, 08035 Barcelona, Spain; 8 Department of Surgery, Hospital Universitari Josep Trueta, 17007 Girona, Spain; 9 Department of Surgery, Hospital Universitari Arnau de Vilanova, 25198 Lleida, Spain; 10 Department of Surgery, Hospital de la Santa Creu i Sant Pau, Universitat Autònoma de Barcelona, 08025 Barcelona, Spain; 11 Department of Surgery, Hospital Universitari Germans Trias i Pujol, Universitat Autònoma de Barcelona, 08916 Badalona, Spain; 12 Department of Surgery, Hospital Universitari Parc Taulí de Sabadell, 08208 Sabadell, Spain; 13 Department of Surgery, Hospital General de Granollers, 08402 Granollers, Spain; 14 Department of Surgery, Hospital Universitari de Tarragona Joan XXIII, 43005 Tarragona, Spain; 15 Department of Surgery, Hospital Universitari de Vic, 08500 Vic, Spain; 16 Department of Surgery, Althaia Xarxa Assistencial Universitària de Manresa, 08243 Manresa, Spain; 17 Faculty of Medicine, Universitat de Vic-Universitat Central de Cataluña (UVIC-UCC), 08500 Vic, Spain; 18 Institut de Recerca i Innovació en Ciències de la Vida i de la Salut a la Catalunya Central (IRIS-CC), 08500 Vic, Spain; 19 Section of Gastrointestinal Surgery, Hospital del Mar, Department of Surgery, Universitat Autònoma de Barcelona, Hospital del Mar Medical Research Institute (IMIM), 08003 Barcelona, Spain; 20 Biostatistics Support and Research Unit, Germans Trias i Pujol Research Institute and Hospital (IGTP), 08916 Barcelona, Spain

**Keywords:** gastric cancer, preoperative biomarker, preoperative inflammation score, preoperative immune score, preoperative nutritional score

## Abstract

**Simple Summary:**

The aim of this study was to evaluate 18 different preoperative immune, inflammatory, and nutritional scores and their best cut-off values as predictors of poorer overall survival (OS) and disease-free survival (DFS) in patients who underwent curative gastrectomy for gastric adenocarcinoma. This is a retrospective observational multicentre study based on data of the Spanish EURECCA Esophagogastric Cancer Registry. Time-dependent Youden index and log-rank test were used to obtain the best cut-offs of preoperative biomarkers for OS and DFS. The most relevant preoperative biomarkers of poorer OS and DFS were high neutrophil-to-lymphocyte ratio (NLR), high monocyte systemic inflammation index (moSII) (for stages I and III), and low prognostic nutritional index (PNI) (regardless tumour stage).

**Abstract:**

**Background:** The aim of this study was to evaluate different preoperative immune, inflammatory, and nutritional scores and their best cut-off values as predictors of poorer overall survival (OS) and disease-free survival (DFS) in patients who underwent curative gastric cancer resection. **Methods:** This was a retrospective observational multicentre study based on data of the Spanish EURECCA Esophagogastric Cancer Registry. Time-dependent Youden index and log-rank test were used to obtain the best cut-offs of 18 preoperative biomarkers for OS and DFS. An adjusted Cox model with variables selected by bootstrapping was used to identify the best preoperative biomarkers, which were also analysed for every TNM stage. **Results:** High neutrophil-to-lymphocyte ratio (NLR), high monocyte systemic inflammation index (moSII), and low prognostic nutritional index (PNI) were identified as independent predictors of poor outcome: NLR > 5.91 (HR:1.73; 95%CI [1.23–2.43]), moSII >2027.12 (HR:2.26; 95%CI [1.36–3.78]), and PNI >40.31 (HR:0.75; 95%CI [0.58–0.96]) for 5-year OS and NLR > 6.81 (HR:1.75; 95%CI [1.24–2.45]), moSII > 2027.12 (HR:2.46; 95%CI [1.49–4.04]), and PNI > 40.31 (HR:0.77; 95%CI [0.60,0.97]) for 5-year DFS. These outcomes were maintained in the whole cohort for NLR and moSII (*p* < 0.05) but not in stage II and for PNI in all tumoral stages. The associations of NLR-PNI and moSII-PNI were also a relevant prognostic factor for OS. **Conclusions:** High NLR, high moSII (for stages I and III), and low PNI (regardless of tumour stage) were the most promising preoperative biomarkers to predict poor OS and DFS in gastric cancer patients treated with curative intent.

## 1. Introduction

Gastric cancer is one of the most common malignancies worldwide, with few improvements in overall long-term results in recent decades [1,2,3]. Surgery remains the main treatment in candidates for curative intent, but perioperative adjuvant treatment has been shown to improve survival in patients with non-metastatic locally advanced tumours [4]. Prognostic evaluation of resectable gastric cancer is mainly based on histological assessment of the resected specimen (pTNM) [5]. However, a reliable preoperative prognostic evaluation is crucial in order to establish the most adequate multimodal personalised treatment. Preoperative tumour staging based on endoscopy, endoscopic ultrasound, computed tomography, or positron emission tomography could have some limitations [6,7]. Immune, inflammatory, and nutritional markers determined before surgery in peripheral blood cells have been demonstrated to correlate with long-term prognosis [8], the risk of postoperative complications [9], and histological response to neoadjuvant treatment [10]. Moreover, several of these markers have been proven to define different prognostic subgroups, being associated with a poor time-survival events for the same pathological TNM stages [11,12]. 

Several preoperative immunity, inflammation, nutritional, and combined markers have been identified as independent predictors of overall survival (OS) and disease-free survival (DFS) in recent studies, the most relevant being: neutrophil-to-lymphocyte ratio (NLR), lymphocyte-to-monocyte ratio (LMR), Glasgow prognostic score (GPS), prognostic nutritional index (PNI), systemic inflammation index (SII), and platelet-to-lymphocyte ratio (PLR) [12,13,14,15,16,17]. However, there is no consensus on which of them is more relevant, probably because most studies are based on short case series and use different cut-off values, making it difficult to compare them and to standardise their routine application. Moreover, most studies come from Asiatic cohorts, which are known to present different treatment responses and outcomes compared to Western populations [11,12,16,17].

The aim of the present study was to evaluate various preoperative immune, inflammatory, and nutritional scores in a Spanish multicentre database registry of patients undergoing gastric cancer resection with curative intent in order to identify the best cut-off values for each score by a time-dependent statistical method and to select the most relevant predictors of poor OS and DFS.

## 2. Methods

### 2.1. Source of Data

This study involves a multicentre, retrospective analysis, based on a prospectively maintained population-based online database (Spanish EURECCA Esophagogastric Cancer Registry, SEEGCR), in which all patients with primary gastric cancer undergoing surgery with curative intent in 15 public hospitals are included. The SEEGCR is an audited database with high completeness and accuracy [18]. 

### 2.2. Ethics

The SEEGCR was approved by the Ethical Committee of Hospital del Mar as a promotor centre and by the Ethical Committee of each centre. All the patients signed an informed consent form before their inclusion in the registry and authorised the use of their data for research purposes. Moreover, the present study was approved by the scientific committee of SEEGCR and was conducted following the STROBE guidelines for reporting observational studies [19].

### 2.3. Patients

All consecutive patients with primary gastric or esophagogastric junction adenocarcinoma (Siewert 2 or 3) undergoing only gastric resection with curative intent (R0) between January 2014 and December 2018 were selected for the study. The data collection and the statistical analysis were started in October 2021. Exclusion criteria were: (1) age under 18 years; (2) chronic inflammatory disease: obesity (body mass index > 40 kg/m^2^), liver cirrhosis, chronic pancreatitis, or inflammatory bowel disease; (3) autoimmune or haematological disease; (4) R1 and R2 resections; (5) intraoperative finding of peritoneal metastases or liver metastasis; (6) palliative surgery; and (7) missing follow-up or preoperative or postoperative white blood cell count data.

### 2.4. Data Collection

The following clinicopathological data were systematically collected: age, gender, American Society of Anesthesiologists (ASA) grade classification, Eastern Cooperative Oncology Group (ECOG) performance status, Charlson comorbidity index, tumour location (proximal, middle, distal third, or gastric linitis), type of gastrectomy (total, subtotal, or other), type of lymphadenectomy according to the Japanese Gastric Cancer Guidelines 2017 [20], neoadjuvant and adjuvant treatment, pathological TNM (according to 7th edition TNM classification [5]) and Lauren classification (intestinal, diffuse, or mixed) [21], severity of postoperative complications occurring during the first 30 days after surgery registered according to both the Clavien-Dindo (C-D) classification [22] and the comprehensive complication index [23]. Postoperative complications were grouped as minor (C-D grade I–II); major (C-D grade III–IV); and mortality (C-D grade V). They were also classified as infectious or non-infectious [24].

Preoperative blood tests were registered in all patients (even in those patients receiving neoadjuvant treatment) using the last blood test before surgery within the previous two weeks: tumoral markers (CEA and CA 19-9), leucocytes, neutrophils, lymphocytes, monocytes, albumin, platelets, haemoglobin, and C-reactive protein (CRP). Based on these preoperative blood test results the following prognostic scores were calculated: (1) immune scores: neutrophil-to-lymphocyte ratio (NLR); lymphocyte-to-monocyte ratio (LMR); monocyte-to-lymphocyte ratio (MLR); systemic inflammation index (SII): platelets × neutrophil/lymphocyte; platelet-to-lymphocyte ratio (PLR); haematological inflammatory index (HII): [(platelets/neutrophil)/lymphocyte] × 100; derived NLR (dNLR) [25]: neutrophil/(leucocyte − neutrophil); monocyte SII (moSII): platelets × neutrophil/lymphocyte × MLR. (2) Inflammatory scores: CRP-to-lymphocyte ratio (CLR); CRP-to-albumin ratio (CAR); prognostic index (PI): patients with elevated CRP (>10 mg/L) and white blood cells (>11 × 10^9^/L) were allocated a score of 2, and patients with one or neither were allocated a score of 1 or 0, respectively; Glasgow prognostic score (GPS): patients with both hypoalbuminaemia (<35 g/L) and an elevated CRP (>10 mg/L) were allocated a score of 2, and patients with one or neither were allocated a score of 1 or 0, respectively. (3) Nutritional scores: prognostic nutritional index (PNI): 10 × albumin (g/dl) + 0.005 × lymphocytes; advanced lung cancer inflammatory index (ALI): body mass index (Kg/m^2^) × albumin (g/dL)/NLR; platelet-to-albumin ratio (PAR); neutrophil-to-albumin ratio (NAR); combined albumin and NLR (COA-NLR) [26]: patients with both hypoalbuminaemia (<35 g/L) and an elevated NLR (>2.3) were allocated a score of 2, and patients with one or neither were allocated a score of 1 or 0, respectively; systemic inflammatory score (SIS): patients with hypoalbuminaemia (<40 g/L) and LMR < 4.44 were allocated a score of 2, and patients with one or neither were allocated a score of 1 or 0, respectively. As CPR and albumin were not routinely recorded in all centres, scores including these parameters were analysed only for patients with available data, as long as there were at least 375 patients included in each score in order to obtain acceptable estimations.

Postoperative follow-up was performed according to each hospital protocol, mostly following international guidelines [20,27,28], and recording tumour relapse (local, nodal, carcinomatosis or systemic different than carcinomatosis) and death.

### 2.5. Statistical Analysis

Data are shown as frequencies and percentages for categorical variables or as median (interquartile range, IQR) for quantitative variables. OS was calculated from date of surgery until death from any cause or date of last follow-up in living patients. DFS was calculated from date of surgery to date of tumour relapse or death or date of last follow-up in living patients without relapse. 

Two methods were used to compute cut-off values for each preoperative index for OS and DFS: (1) using time-dependent receiver operating characteristic (t-ROC) curves, we selected the value that maximises the Youden index (YI) and (2) we selected the value that most separated the two survival curves according to the log-rank test (LRt). Based on these two cut-offs points, biomarkers were categorised into two groups (low and high risk), if the cut-offs had a similar value, or into three groups (low, intermediate, and high risk) if they did not. Association of these categories with OS and DFS was assessed with the Kaplan–Meier method and compared using the log-rank test.

The best-performing biomarkers were selected and raw and adjusted Cox models were estimated for OS and DFS. Bootstrapping was used to select the variables to include in the adjusted model. Data were sampled 1000 times by bootstrapping. For each bootstrap sample, a Cox model was fitted using backward elimination. Variables retained in more than 75% of the models (age, tumoral stage, tumour location, Charlson index, CEA, and BMI) were candidates for inclusion as adjustment variables in the final model.

Two Cox models were then calculated for OS and for DFS: (1) model with only the biomarker as the explanatory variable (dichotomous if the two cut-off values were similar and in three categories if the two cut-off values were different), (2) model 1 adjusted by the variables obtained in bootstrapping, plus tumoral stage (pTMN) and neoadjuvant therapy (selected by clinical criteria). The hazard ratio (HR) and the 95% confidence interval (CI) for each biomarker category were reported.

Finally, an analysis with the most relevant biomarkers was performed within every TNM stage. All analyses were performed with a two-sided significance level of 0.05 using R software version 4.3.0 [29]. The main R packages used for data management and analysis were dplyr, compareGroups, gtsummary, and survival-ROC.

## 3. Results

### 3.1. Descriptive Analysis

**Figure 1:**
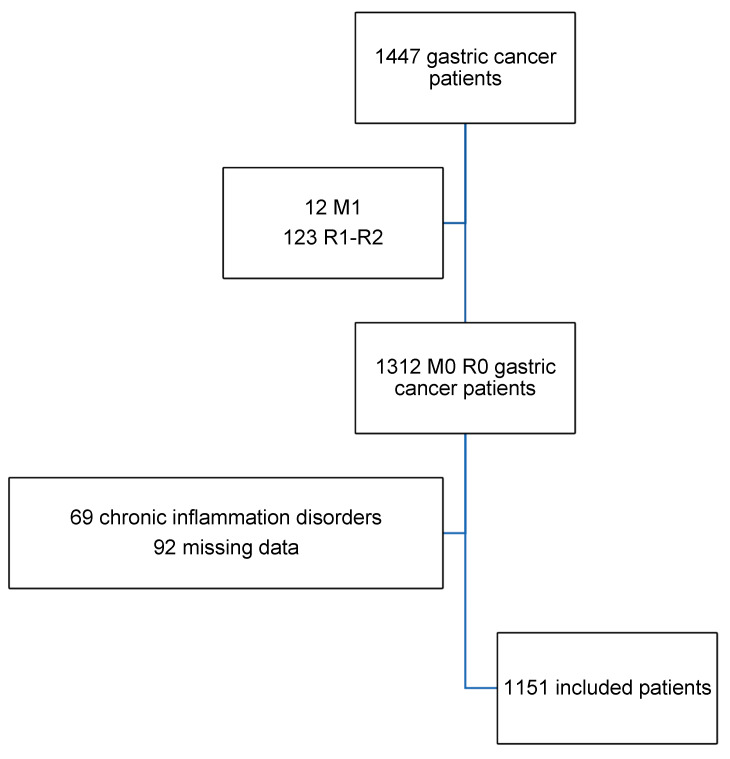
Flow chart of the study population.

During the study period, 1447 patients were registered, of whom 346 presented one or more exclusion criteria, leaving 1151 patients for analysis (Figure 1). The mean age was 69.5 years and most of the patients were male (61.9%) and had a good performance status, the Charlson index 2 and ECOG 1 being the most common. Subtotal gastrectomy (59.1%) and D2 lymphadenectomy (48.2%) were the most frequent surgical procedures. Major postoperative complications occurred in 213 (41%) patients, while 222 (19.5%) patients presented infectious complications. Detailed information about tumour location, type of surgery, tumoral stage, and postoperative results is depicted in Table 1. Table 2 shows the median and IQR values for all the preoperative immune, inflammatory, and nutritional scores. Preoperative indexes expressed in progressive categories are listed with their frequencies. The median follow-up of the whole cohort was 45 months (0–105). 

### 3.2. Excluded Preoperative Biomarkers

Cut-off values for each score, calculated to predict both OS and DFS, are listed in Supplementary Tables S1–S4. The following biomarkers were excluded because no statistical significance was identified in the raw Cox model: dNLR, HII, PAR, NAR, CAR, and CLR. SII was also excluded because of its similarity to moSII, the latter having more relevant results. After adjusted analysis, the following indexes were also excluded: ALI, GPS, SIS, PI, PLR, LMR, and COA-NLR. MLR (cut-off > 0.56) remained as an independent prognostic factor for OS (HR 1.74, 95%CI 1.3–2.33; *p*: 0.001) and DFS (HR 1.55, 95%CI 1.17–2.04; *p*: 0.002), but because its similarity to moSII, it was also excluded.

### 3.3. Most Relevant Preoperative Biomarkers

The most relevant preoperative markers for OS and DFS according to adjusted Cox models were NLR, PNI, and moSII. Cut-off values for OS were: 1.99 (YI) and 5.91 (LRt) for NLR; 40.61 (YI) and 40.31 (LRt) for PNI; 116.45 (YI) and 2027.12 (LRt) for moSII. Cut-off values for DFS were: 2.03 (YI) and 6.81 (LRt) for NLR; 40.21 (YI) and 40.31 (LRt) for PNI; 110.74 (YI) and 2027.12 (LRt) for moSII (Supplementary Figure S1).

**Figure 2:**
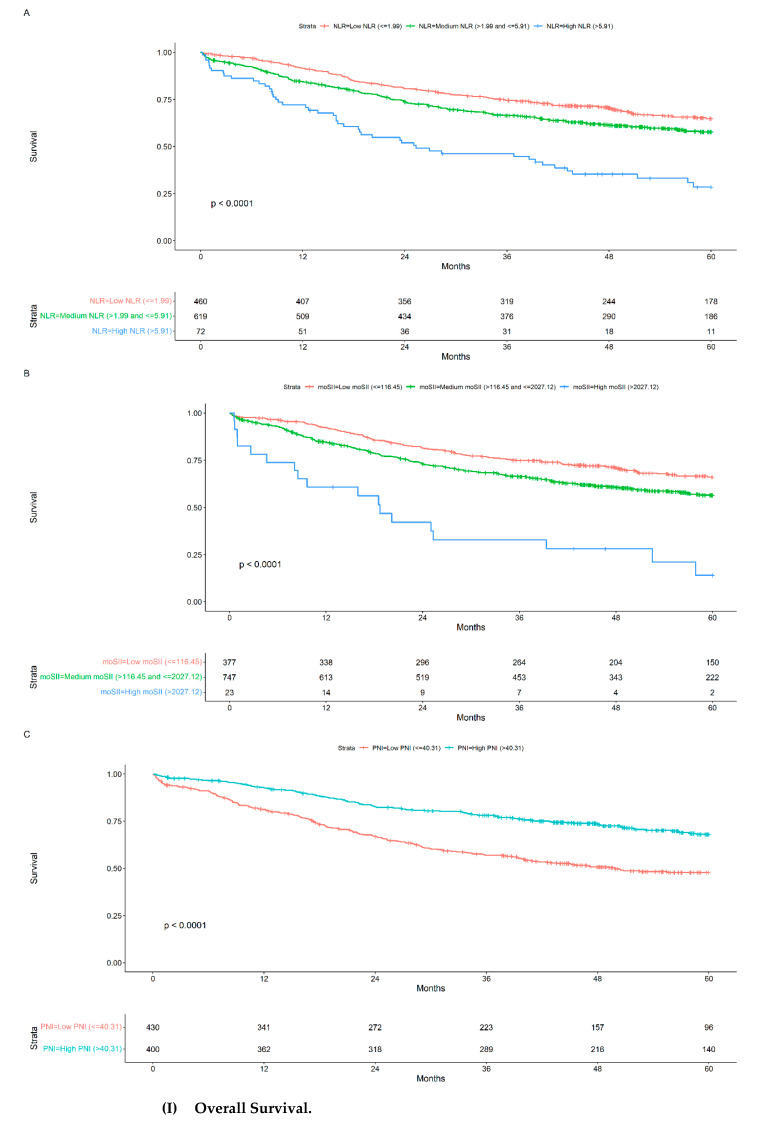

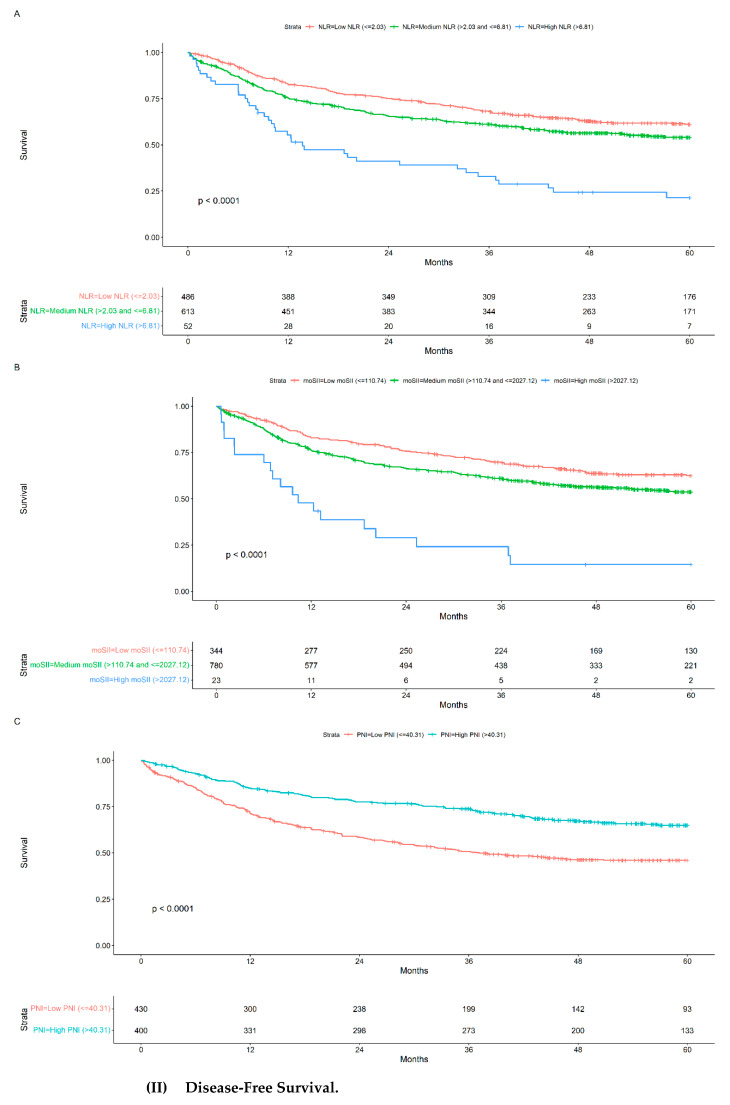
Kaplan–Meier curves for NLR (**A**), moSII (**B**), and PNI (**C**) categories in (**I**,**II**).

OS and DFS were analysed according to the NLR, moSII, and PNI categories. High NLR (>5.91), high moSII (>2027.12), and low PNI (≤40.31) categories were identified as prognostic factors of poor outcome, with OS at 5 years of 28.45%, 14.05%, and 47.78%, respectively (Figure 2I), and DFS at 5 years of 21.34%, 14.05%, and 45.91%, respectively (Figure 2II).

In adjusted analysis, NLR > 5.91 (HR: 1.73; 95% CI [1.23–2.43], *p*: 0.002), moSII > 2027.12 (HR 2.26; 95% CI [1.36–3.78], *p*: 0.002), and PNI > 40.31 (HR: 0.75; 95% CI [0.58–0.96], *p*: 0.023) behaved as independent prognostic factors for OS (Table 3). For DFS, NLR >6.81 (HR: 1.75; 95% CI [1.24–2.45], *p*: 0.001), moSII > 2027.12 (HR: 2.46; 95% CI [1.49–4.04], *p* < 0.001), and PNI > 40.31 (HR: 0.77; 95% CI [0.60, 0.97], *p*: 0.028) were independent prognostic factors (Table 4).

### 3.4. Tumour Stage and Preoperative Biomarkers 

Supplementary Figure S2 shows the Kaplan–Meier curves of NLR, PNI, and moSII within each tumour pathological stage: patients with high NLR or high moSII showed the worst OS and DFS outcomes for stages I and III (*p* < 0.05) and also patients with low PNI for stages I, II, and III (*p* < 0.05). These results were not obtained in stage II with high NLR or high moSII, where the physical status of the patient (measured by the Charlson index) and age were the most relevant preoperative prognostic factors. Although PLR was excluded in the previous Cox models, it was identified as an independent prognostic factor in stage III tumours (HR 1.5, 95% CI 1.15–1.97; *p*: 0.003).

### 3.5. Combination of Preoperative Biomarkers

The prognostic roles of NLR, PNI, and moSII biomarkers in overall survival were analysed together in an adjusted multivariate Cox regression model. Including the three biomarkers in the same model did not yield statistically significant results and did not improve model performance. However, the association of PNI-NLR or PNI-moSII suggests a better independent prognostic role of both biomarkers in overall survival than each one alone, even after adjustment for age, ECOG, tumour localization, Charlson index, CEA, body mass index, tumour stage, and neoadjuvant therapy (Supplementary Table S5).

## 4. Discussion

The present study evaluated and compared different preoperative immune, inflammatory, and nutritional biomarkers described in the literature in order to identify the most relevant prognostic predictors of poor OS and DFS for patients with resectable gastric cancer. Time-dependent adjusted analysis showed that high NLR and low PNI were the preoperative categories that more strongly correlate with the most unfavourable long-term prognosis after surgery. These findings are consistent with previous studies that reported the usefulness of NLR [30] and PNI [31] as preoperative prognostic markers, regardless of tumour stage. However, detailed comparison of the present study’s results with previous data is hindered by the wide heterogeneity in the definition of the categories of biomarkers, mainly because many of the studies were based on small case series and calculated static cut-off values [14,17,32]. The present study identifies the thresholds that best divided patients with better and worst outcomes, taking into account the evolution of the disease (time-dependent analysis) in a large homogeneous cohort. To our knowledge, this is the first study to evaluate the time-dependent correlation of the biomarkers with OS and DFS separately. The best cut-off values were the same for both outcomes in the cases of PNI (40.31) but found to be different for NLR in the evaluation of OS (5.91) and DFS (6.81).

The prognostic usefulness of these preoperative biomarkers remained when evaluated within tumour stages. Stage I and stage III gastric cancer patients with high NLR or any tumoral stage (stages I, II, and III) with low PNI presented with worse OS and DFS. This means that patients identified in the same tumour stage may have different evolution depending on their inflammatory and immune activation and their nutritional state. These findings are in line with previous publications in which preoperative inflammatory markers using lymphocyte and monocyte values seem to be the most favourable for discriminating patients with advanced gastric cancer [33]. However, preoperative high NLR was not so useful in patients with intermediate tumour stage (stage II), where the functional status and age seemed to be more relevant for prognosis. These results differ from a previous publication, in which low SII identified differences in survival for stage II gastric cancer patients [16]. Together with NLR and PNI, MLR was also identified as a good estimator of OS and DFS, in accordance with previous studies [34]; and PLR was found to correlate with DFS in stage III patients, also in agreement with previous studies [34,35]. 

In an exploratory analysis, moSII (a combination of the SII and MLR) appears as a new preoperative inflammation index with promising predictive capacity, although this finding should be confirmed in future studies. As for high NLR and low PNI, high moSII behaves as a poorer prognostic index of OS and DFS, maintaining these outcomes in tumour stages I and III. Additionally, the association of immunological and nutritional markers (moSII-PNI) in a Cox model improved prognosis estimation, in consonance with previous studies suggesting that PNI-SII was a promising predictor of chemotherapy response and OS [36]. This suggests the development of clinical prediction models that include these biomarkers as potential predictors of overall survival beyond classical predictors.

An increase in moSII-PNI score indicates a relative decrease in lymphocytes, suggesting that the patient may be immunosuppressed or deficient, thus promoting tumour progression and affecting the patient’s prognosis [36]. The decrease in serum albumin levels has been associated with an increase in proinflammatory cells, in addition to a poorer nutritional status. Also, patients with a state of malnutrition will present with lower immunity, which affects disease progression [37]. 

Although postoperative complications are known to be a prognostic factor [9,24], they were not included in the multivariate analysis because the aim of the present study was to assess preoperative prognostic markers. Previous studies have analysed postoperative complications in relation to preoperative inflammatory markers, showing that they also had an impact on long-term prognosis [24]. The influence of the preoperative biomarkers according to pathological nodal stage has also been described [38] but was not the aim of the present study. Further exploratory analysis would be interesting. However, other preoperative parameters were considered such as neoadjuvant therapy, age, ECOG, tumour localization, Charlson index, CEA, BMI, and tumour stage.

The findings of this study may contribute to a more accurate preoperative assessment and allow a tailored multimodal treatment design, depending on each patient’s risk for tumour relapse. The potential use of prognostic biomarkers in clinical practice requires a previous agreement on their cut-off values for category definition, to which the present study may contribute. In addition, the confirmation that preoperative nutritional, inflammatory, and immune statuses of patients affect their long-term outcome, regardless of their tumour stage, could also have some direct therapeutic implications: some high-risk patients may benefit from preoperative nutritional prehabilitation, together with inflammatory and immune perioperative regulation; other patients at risk may benefit from closer follow-up or this fact should be taken into account when administering neoadjuvant or adjuvant chemotherapy. 

The present study has some limitations, mainly in relation to its observational nature. Although blood test data were prospectively collected in the registry, included parameters depended on each centre’s protocol, so some of them were missing. This limitation tends to be compensated by the high number of patients, the systematic exclusion of the scores with fewer cases, a limited amount of missing information, the multicentre character of the study, and the adjusted statistical analysis. All blood test samples were recorded less than two weeks before the surgery, even in patients with neoadjuvant treatment, as previously reported [39]. Although this may be one of the first studies evaluating most of the known preoperative scores, some others could not be analysed because they use blood test parameters which were not routinely collected in our database, such as fibrinogen, included in systemic inflammatory response index (F-SIRI) [40]; or total cholesterol, needed for the Controlling Nutritional Status (CONUT) [41]. Another limitation could be found in the low number of patients in the analysis of the biomarkers according to tumoral stage. This is because we have identified the subgroup of patients with the worst clinical prognosis and where our findings could achieve more clinical relevance. Nonetheless, these results present a high validity and are comparable due to a statistical process with a high internal validity achieved by bootstrapping. External validation of the model remains to be tested in an independent cohort.

## 5. Conclusions

In conclusion, this study identified high NLR, high moSII (for stages I and III), and low PNI (regardless of tumour stage) as the most promising preoperative biomarkers to predict poor OS and DFS in gastric cancer patients treated with curative intent. Therefore, the nutritional, inflammatory, and immune status of the patient should be considered to predict the long-term prognosis and for designing tailored multimodal treatment and surgical prehabilitation approaches.

## Figures and Tables

**Table 1:** Baseline characteristics and postoperative outcomes.

Baseline Characteristics		*n* (1151)
**Age, Mean (SD)**	69.5 (11.8)	
**Gender**		
Male	713 (61.9%)	
Female	438 (38.1%)	
**Charlson Index, Mean (SD)**	2.96 (1.33)	
**Charlson Index**		
2	572 (49.7%)	
3	305 (26.5%)	
4	139 (12.1%)	
≥5	135 (11.7%)	
**ECOG**		
0	415 (36.1%)	
1	615 (53.4%)	
2	101 (8.8%)	
3	20 (1.7%)	
**ASA**		
1	50 (4.3%)	
2	526 (45.7%)	
3	536 (46.6%)	
4	39 (3.4%)	
**Tumour location**		
1/3 Proximal	150 (13%)	
1/3 Middle	395 (34.3%)	
1/3 Distal	588 (51.1%)	
Other	18 (1.6%)	
**Neoadjuvant treatment**		
No	786 (68.3%)	
Yes	365 (31.7%)	
**Type of gastrectomy**		
Subtotal	680 (59.1%)	
Total	470 (40.8%)	
Other	1 (0.1%)	
**Type of lymphadenectomy**		
D1	196 (17%)	
D1+	397 (34.5%)	
D2	555 (48.2%)	
Other	3 (0.3%)	
**TNM stage**		
1	448 (38.9%)	
2	290 (25.2%)	
3	413 (35.9%)	
**pT**		
0	32 (2.8%)	
1	304 (26.4%)	
2	192 (16.7%)	
3	318 (27.6%)	
4	305 (26.5%)	
**pN**		
0	581 (50.5%)	
1	201 (17.5%)	
2	186 (16.2%)	
3a	125 (10.9%)	
3b	58 (5.04%)	
**Lauren classification**		1094
Diffuse	326 (29.7%)	
Intestinal	622 (56.8%)	
Mixed	146 (13.3%)	
**Adjuvant treatment**		
No	610 (53%)	
Yes	539 (47%)	
**Postoperative Outcomes**		
Postoperative complications		
No	635 (55.2%)	
Yes	516 (44.8%)	
Clavien–Dindo classification		516
Type I–II	303 (58.7%)	
Type III–V	213 (41.3%)	
**CCI**	15.2 [0–21]	
Infectious complications		
No	929 (80.7%)	
Yes	222 (19.3%)	
Mortality		
No	679 (59.0%)	
Yes	472 (41.0%)	
Tumour relapse		
No	802 (69.7%)	
Yes	349 (30.3%)	
**Type of relapse**		
Local	35 (3.0%)	
Lymph node	52 (4.5%)	
Peritoneal metastasis	82 (7.1%)	
Systemic	116 (10.1%)	
Combined	64 (5.6%)	

SD = standard deviation; ECOG = Eastern Cooperative Oncology Group; ASA = American Society of Anesthesiologists; CCI = comprehensive complication index.

**Table 2:** Descriptive results of preoperative biomarkers.

Preoperative Markers	*n*	Median (IQR)
**CEA**	1151	4.40 [1–3]
**CA 19.9**	922	55.02 [5–21]
**NLR**	1151	2.23 [1.63;3.25]
**LMR**	1151	2.93 [2.19;3.80]
**PLR**	1151	135 [102;186]
**MLR**	1151	0.34 [0.26;0,46]
**dNLR**	1151	10.5 [6.71;15.4]
**SII**	1147	524 [347;832]
**moSII**	1147	173 [96.9;348]
**HII**	1151	35.0 [23.6;51.2]
**PNI**	830	40.0 [36.9;43.1]
**ALI**	813	45.1 [30.1;67.0]
**PAR**	830	6.09 [4.79;7.65]
**NAR**	830	0.10 [0.08;0.14]
**CAR**	375	0.55 [0.18;1.91]
**CLR**	425	1.39 [0.45;4.93]
**COA-NLR**	829	
0		375 (45.2%)
1		343 (41.4%)
2		111 (13.4%)
**GPS**	375	
0		253 (67.5%)
1		85 (22.7%)
2		37 (9.87%)
**SIS**	830	
0		122 (14.7%)
1		708 (85.3%)
2		0 (0%)
**PI**	424	
0		321 (75.7%)
1		91 (21.5%)
2		12 (2.83%)

NLR = neutrophil-to-lymphocyte ratio; LMR = lymphocyte-to-monocyte ratio; PLR = platelet-to-lymphocyte ratio; MLR = monocyte-to-lymphocyte ratio; dNLR = derived neutrophil-to-lymphocyte ratio; SII = systemic inflammatory index; moSII = monocyte systemic inflammatory index; HII = haematological inflammatory index; PNI = prognostic nutritional index; ALI = advanced lung cancer inflammatory index; PAR = platelet-to-albumin ratio; NAR = neutrophil-to-albumin ratio; CAR = C-reactive-protein-to-albumin ratio; CLR = C-reactive-protein-to-lymphocyte ratio; COA-NLR = combined albumin concentration and neutrophil-to-lymphocyte ratio; GPS = Glasgow prognostic score; SIS = systemic inflammation score; PI = prognostic index; IQR: interquartile range.

**Table 3:** Raw and adjusted Cox models for NLR, moSII, and PNI for overall survival.

	Raw Model			Adjusted Model *		
	HR	95% CI	*p*-Value	HR	95% CI	*p*-Value
**NLR**						
Low NLR (≤1.99)	Ref			Ref		
Medium NLR (>1.99 and ≤5.91)	1.33	1.09, 1.64	0.006	1.10	0.89, 1.36	0.4
High NLR (>5.91)	2.94	2.12, 4.07	<0.001	1.73	1.23, 2.43	0.002
**moSII**						
Low moSII (≤116.45)	Ref			Ref		
Medium moSII (>116.45 and ≤2027.12)	1.43	1.15, 1.77	0.001	1.20	0.96, 1.50	0.11
High moSII (>2027.12)	4.35	2.64, 7.15	<0.001	2.26	1.36, 3.78	0.002
**PNI**						
Low PNI (≤40.31)	Ref			Ref		
High PNI (>40.31)	0.48	0.38, 0.61	<0.001	0.75	0.58, 0.96	0.023

HR = hazard ratio, CI = confidence interval, NLR = neutrophil-to-lymphocyte ratio, moSII = monocyte systemic inflammation index; PNI = prognostic nutritional index. * All models adjusted by age, ECOG, tumour localization, Charlson index, CEA, body mass index, tumour stage, and neoadjuvant therapy.

**Table 4:** Raw and adjusted Cox models for NLR, moSII, and PNI for disease free survival.

	Raw Model			Adjusted Model *		
	HR	95% CI	*p*-Value	HR	95% CI	*p*-Value
**NLR**						
Low NLR (≤2.03)	Ref			Ref		
Medium NLR (>2.03 and ≤6.81)	1.29	1.07, 1.57	0.008	1.11	0.91, 1.35	0.3
High NLR (>6.81)	3.00	2.12, 4.25	<0.001	2.00	1.40, 2.85	<0.001
**mSII**						
Low moSII (≤110.74)	Ref			Ref		
Medium moSII (>110.74 and ≤2027.12)	1.35	1.10, 1.66	0.004	1.18	0.95, 1.46	0.13
High moSII (>2027.12)	4.26	2.62, 6.91	<0.001	2.43	1.48, 4.01	<0.001
**PNI**						
Low PNI (≤40.31)	Ref			Ref		
High PNI (>40.31)	0.52	0.42, 0.64	<0.001	0.77	0.61, 0.98	0.032

HR = hazard ratio, CI = confidence interval, NLR = neutrophil-to-lymphocyte ratio, moSII = monocyte systemic inflammation index; PNI = prognostic nutritional index. * All models adjusted by age, ECOG, tumour localization, Charlson index, CEA, body mass index, tumour stage, and neoadjuvant therapy.

## Data Availability

Study data can be made available upon reasonable request to the corresponding author.

## Supplementary Materials

The following supporting information can be downloaded at: https://www.mdpi.com/article/10.3390/cancers16122188/s1, Table S1: Cut-offs for mortality according to Youden Index method for all biomarkers, sample size of the two resulting groups and HR and 95% CI of the Cox model with the dichotomized biomarker. Table S2: Cut-offs for mortality according to Log-rank method for all biomarkers, sample size of the two resulting groups and HR and 95% CI of the Cox model with the dichotomized biomarker. Table S3: Cut-offs for disease-free survival according to Youden Index method for all biomarkers, sample size of the two resulting groups and HR and 95% CI of the Cox model with the dichotomized biomarker. Table S4: Cut-offs for disease-free survival according to Log-rank method for all biomarkers, sample size of the two resulting groups and HR and 95% CI of the Cox model with the dichotomized biomarker. Table S5: Multivariant Cox regression models to analyze the association of NLR, moSII and PNI on overall survival. Figure S1. Cut-off values for NLR (A), moSII (B) and PNI (C) according to Youden Index method (blue line) and log-rank test method (orange line). Figure S2: Kaplan-Meier curves for NLR, moSII and PNI categories according to tumoral stage based on TNM classification.
