# Supplementary material for: Prognostic Relevance of Preoperative Immune, Inflammatory, and Nutritional Biomarkers in Patients Undergoing Gastrectomy for Resectable Gastric Adenocarcinoma: An Observational Multicentre Study
Source: Cancers (Basel). 2024 Jun 11;16(12):2188. doi: 10.3390/cancers16122188 (PMC11201927; doi:10.3390/cancers16122188)
Supplement: Supplementary file 1 [file cancers-16-02188-s001.zip › cancers-3052825-supplementary.pdf]

Supplementary Table S1: Cut-offs for mortality according to Youden Index method for all biomarkers, sample size of the two resulting groups and HR and 95% CI of the Cox model with the dichotomized biomarker.

Biomarker	Cut-off	N group low	N group high	HR	CI 95%	p-value
NLR	1.99	460	691	1.18	[0.97; 1.44]	0.091
LMR	1.94	200	951	0.74	[0.6; 0.92]	0.007
PLR	141.22	620	531	1.21	[1.01; 1.46]	0.037
MLR	0.27	338	813	1.28	[1.03; 1.58]	0.024
dNLR	13.33	766	385	1.06	[0.87; 1.28]	0.553
SII	630.00	704	443	1.16	[0.96; 1.39]	0.127
moSII	116.45	377	770	1.25	[1.02; 1.54]	0.031
HII	41.87	728	423	1.02	[0.84; 1.23]	0.853
PNI	40.61	440	390	0.73	[0.58; 0.92]	0.008
ALI	38.98	326	487	0.79	[0.63; 0.98]	0.030
PAR	5.00	245	585	1.15	[0.9; 1.47]	0.253
NAR	0.11	519	311	1.23	[0.99; 1.53]	0.062
CAR	0.51	179	196	1.12	[0.82; 1.54]	0.469
CLR	1.87	242	183	1.12	[0.84; 1.51]	0.429

HR = Hazard Ratio, CI = Confidence Interval, NLR= neutrophil-to-lymphocyte ratio; LMR= lymphocyte-to-monocyte ratio; PLR= platelets-to-lymphocyte ratio; MLR= monocyte-to-lymphocyte ratio; dNLR= derived neutrophil-to-lymphocyte ratio; SII= Systemic inflammatory index; moSII= monocyte-Systemic inflammatory index; HII= Haematological Inflammatory Index; PNI= Prognostic Nutritional Index; ALI= advanced lung cancer inflammatory index; PAR= platelets-to-albumin ratio; NAR= neutrophil-to-albumin ratio; CAR = C-reactive protein-to-albumin ratio; CLR = C-reactive protein-to-lymphocyte ratio; COA-NLR= combining albumin concentration and neutrophil-to-lymphocyte ratio; GPS = Glasgow Prognostic Score; SIS = Systemic Inflammation Score; PI = Prognostic Index.

Supplementary Table S2: Cut-offs for mortality according to Log-rank method for all biomarkers, sample size of the two resulting groups and HR and 95% CI of the Cox model with the dichotomized biomarker.

Biomarker	Cut-off	N group low	N group high	HR	CI 95%	p-value
NLR	5.91	1079	72	1.70	[1.26; 2.3]	<0.001
LMR	1.76	154	997	0.65	[0.52; 0.82]	<0.001
PLR	134.39	575	576	1.26	[1.05; 1.52]	0.013
MLR	0.56	994	157	1.53	[1.22; 1.92]	<0.001
dNLR	20.53	1020	131	1.12	[0.86; 1.47]	0.392
SII	770.12	818	329	1.18	[0.97; 1.44]	0.092
moSII	2027.12	1124	23	2.06	[1.28; 3.31]	0.003
HII	94.19	1088	63	0.70	[0.43; 1.12]	0.132
PNI	40.31	429	401	0.72	[0.57; 0.91]	0.007
ALI	22.61	123	690	0.74	[0.57; 0.97]	0.026
PAR	9.10	718	112	1.15	[0.87; 1.52]	0.343
NAR	0.15	667	163	1.25	[0.98; 1.6]	0.074
CAR	25.01	365	10	1.76	[0.85; 3.64]	0.128
CLR	39.02	404	21	1.51	[0.88; 2.59]	0.131

HR = Hazard Ratio, CI = Confidence Interval, NLR= neutrophil-to-lymphocyte ratio; LMR= lymphocyte-to-monocyte ratio; PLR= platelets-to-lymphocyte ratio; MLR= monocyte-to-lymphocyte ratio; dNLR= derived neutrophil-to-lymphocyte ratio; SII= Systemic inflammatory index; moSII= monocyte-Systemic inflammatory index; HII= Haematological Inflammatory Index; PNI= Prognostic Nutritional Index; ALI= advanced lung cancer inflammatory index; PAR= platelets-to-albumin ratio; NAR= neutrophil-to-albumin ratio; CAR = C-reactive protein-to-albumin ratio; CLR = C-reactive protein-to-lymphocyte ratio; COA-NLR= combining albumin concentration and neutrophil-to-lymphocyte ratio; GPS = Glasgow Prognostic Score; SIS = Systemic Inflammation Score; PI = Prognostic Index.

Supplementary Table S3: Cut-offs for disease-free survival according to Youden Index method for all biomarkers, sample size of the two resulting groups and HR and 95% CI of the Cox model with the dichotomized biomarker.

Biomarker	Cut-off	N group low	N group high	HR	CI 95%	p-value
NLR	2.03	484	667	1.17	[0.97; 1.41]	0.099
LMR	2.01	229	922	0.78	[0.63; 0.96]	0.018
PLR	140.71	618	533	1.28	[1.07; 1.53]	0.008
MLR	0.27	338	813	1.20	[0.98; 1.48]	0.078
dNLR	10.86	601	550	0.93	[0.77; 1.11]	0.425
SII	703.17	775	372	1.22	[1.01; 1.47]	0.039
moSII	110.74	344	803	1.24	[1.01; 1.52]	0.044
HII	26.93	374	777	1.14	[0.94; 1.39]	0.176
PNI	40.21	429	401	0.74	[0.59; 0.94]	0.011
ALI	38.60	320	493	0.80	[0.65; 1]	0.045
PAR	5.00	245	585	1.19	[0.93; 1.52]	0.161
NAR	0.11	495	335	1.16	[0.94; 1.43]	0.179
CAR	0.46	162	213	1.06	[0.78; 1.45]	0.706
CLR	0.81	155	270	1.06	[0.77; 1.44]	0.733

HR = Hazard Ratio, CI = Confidence Interval, NLR= neutrophil-to-lymphocyte ratio; LMR= lymphocyte-to-monocyte ratio; PLR= platelets-to-lymphocyte ratio; MLR= monocyte-to-lymphocyte ratio; dNLR= derived neutrophil-to-lymphocyte ratio; SII= Systemic inflammatory index; moSII= monocyte-Systemic inflammatory index; HII= Haematological Inflammatory Index; PNI= Prognostic Nutritional Index; ALI= advanced lung cancer inflammatory index; PAR= platelets-to-albumin ratio; NAR= neutrophil-to-albumin ratio; CAR = C-reactive protein-to-albumin ratio; CLR = C-reactive protein-to-lymphocyte ratio; COA-NLR= combining albumin concentration and neutrophil-to-lymphocyte ratio; GPS = Glasgow Prognostic Score; SIS = Systemic Inflammation Score; PI = Prognostic Index.

Supplementary Table S4: Cut-offs for disease-free survival according to Log-rank method for all biomarkers, sample size of the two resulting groups and HR and 95% CI of the Cox model with the dichotomized biomarker.

Biomarker	Cut-off	N group low	N group high	HR	CI 95%	p-value
NLR	6.81	1099	52	1.99	[1.43; 2.77]	<0.001
LMR	1.76	154	997	0.68	[0.54; 0.85]	<0.001
PLR	134.39	575	576	1.32	[1.1; 1.58]	0.003
MLR	0.56	994	157	1.47	[1.17; 1.85]	<0.001
dNLR	2.73	25	1126	2.21	[0.92; 5.35]	0.077
SII	770.12	818	329	1.24	[1.02; 1.5]	0.031
moSII	2027.12	1124	23	2.28	[1.43; 3.63]	<0.001
HII	94.19	1088	63	0.66	[0.41; 1.06]	0.087
PNI	40.31	429	401	0.74	[0.59; 0.94]	0.011
ALI	22.61	123	690	0.80	[0.62; 1.05]	0.102
PAR	9.10	718	112	1.17	[0.88; 1.55]	0.272
NAR	0.19	769	61	1.26	[0.89; 1.77]	0.186
CAR	25.01	365	10	1.81	[0.87; 3.76]	0.11
CLR	39.02	404	21	1.53	[0.9; 2.61]	0.118

HR = Hazard Ratio, CI = Confidence Interval, NLR= neutrophil-to-lymphocyte ratio; LMR= lymphocyte-to-monocyte ratio; PLR= platelets-to-lymphocyte ratio; MLR= monocyte-to-lymphocyte ratio; dNLR= derived neutrophil-to-lymphocyte ratio; SII= Systemic inflammatory index; moSII= monocyte-Systemic inflammatory index; HII= Haematological Inflammatory Index; PNI= Prognostic Nutritional Index; ALI= advanced lung cancer inflammatory index; PAR= platelets-to-albumin ratio; NAR= neutrophil-to-albumin ratio; CAR = C-reactive protein-to-albumin ratio; CLR = C-reactive protein-to-lymphocyte ratio; COA-NLR= combining albumin concentration and neutrophil-to-lymphocyte ratio; GPS = Glasgow Prognostic Score; SIS = Systemic Inflammation Score; PI = Prognostic Index.

Supplementary Figure S1. Cut-off values for NLR (Figure A), moSII (Figure B) and PNI (Figure C) according to Youden Index method (blue line) and log-rank test method (orange line).

a) Overall survival

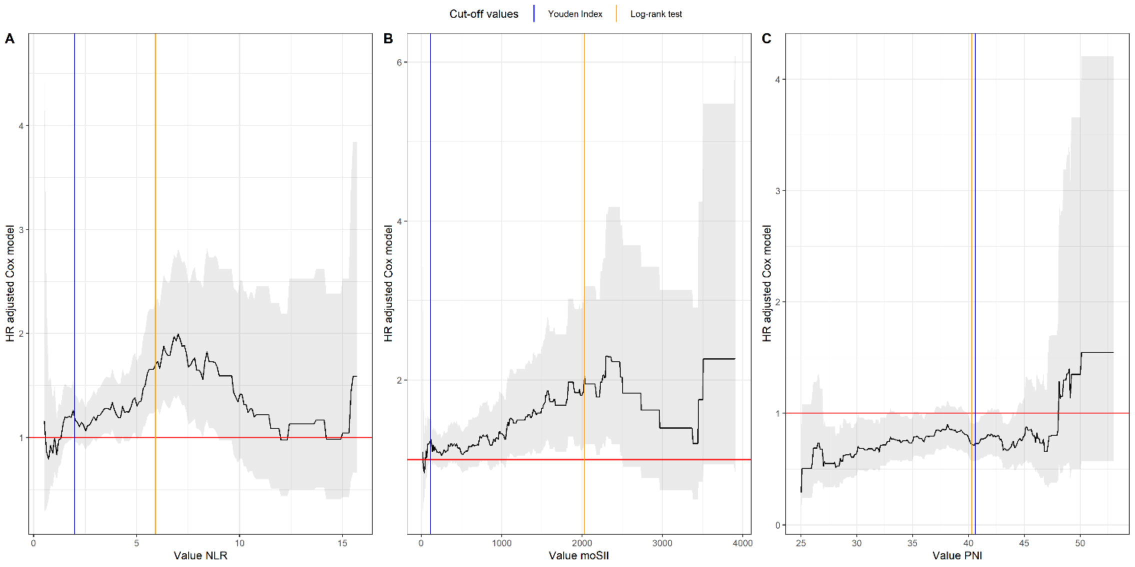

b) Disease free survival

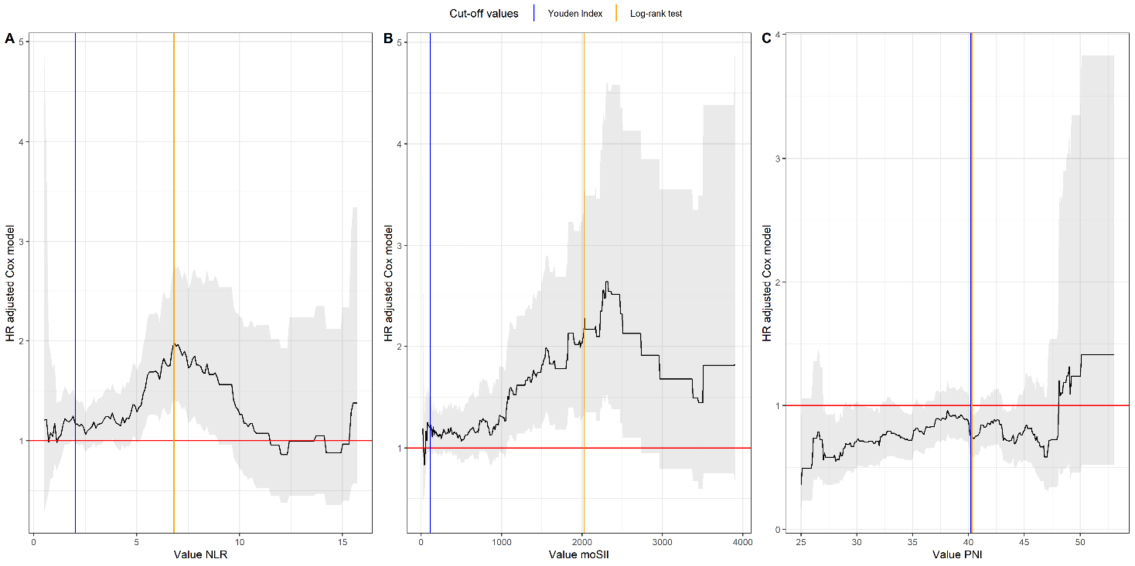

Supplementary Figure S2: Kaplan-Meier curves for NLR, moSII and PNI categories according to tumoral stage based on TNM classification.

a) Overall survival for NLR

Stage I

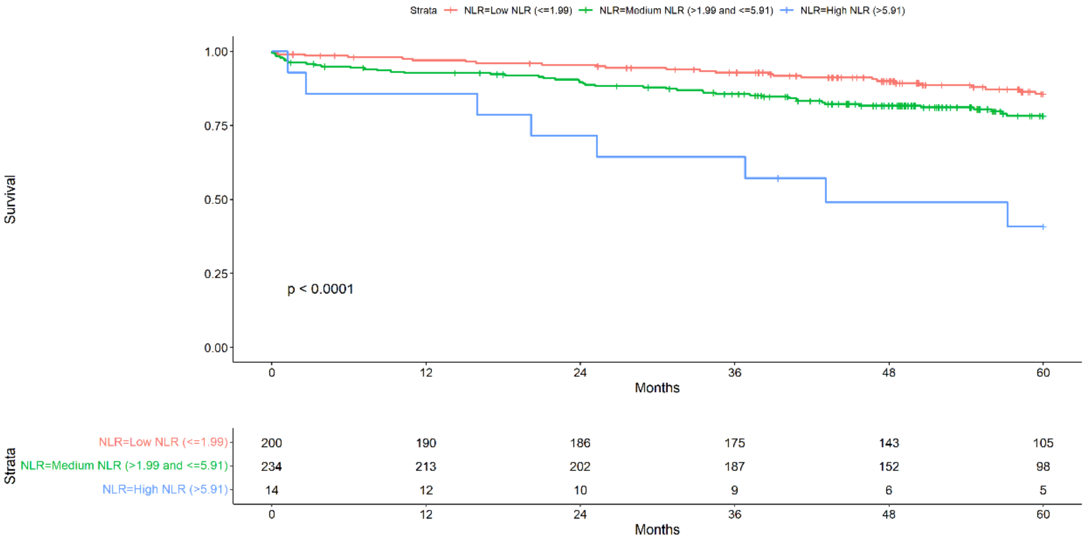

Stage II

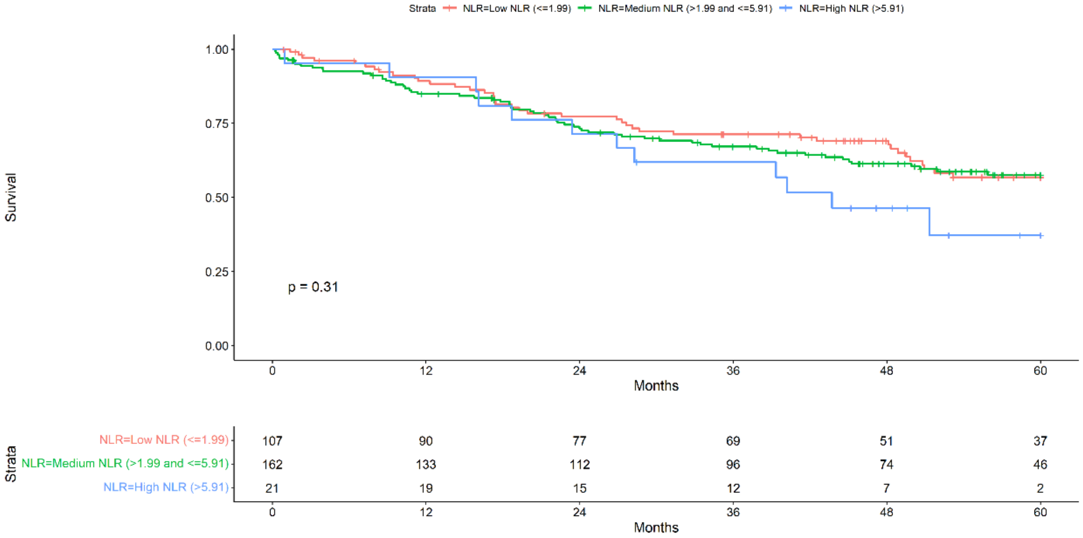

Stage III

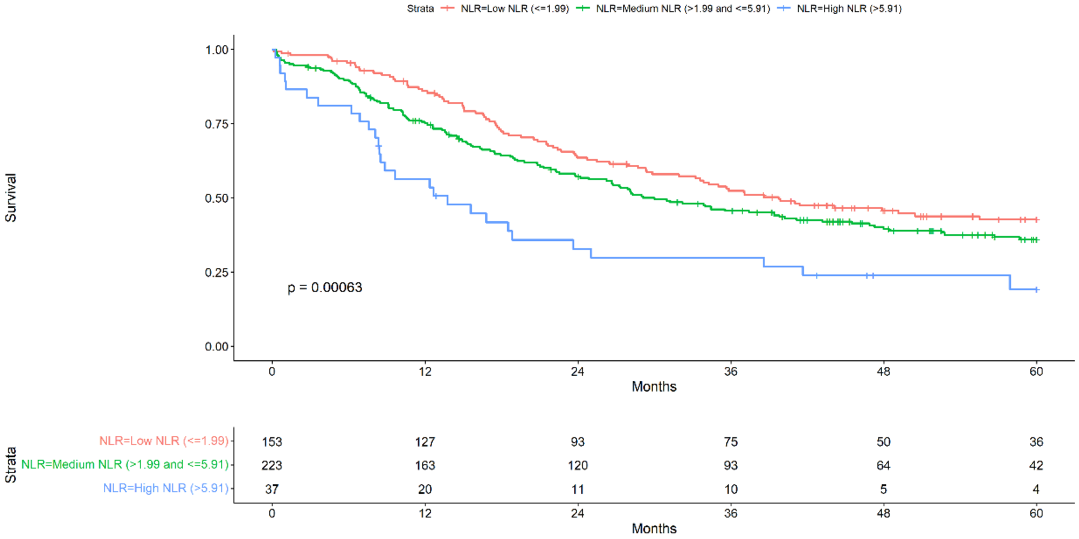

b) Overall survival for moSII

Stage I

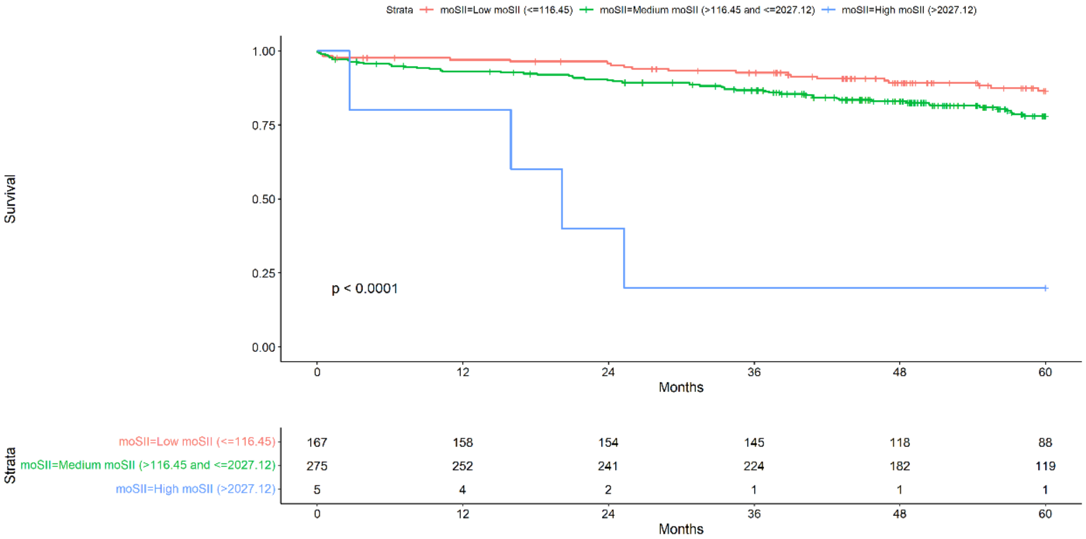

Stage II

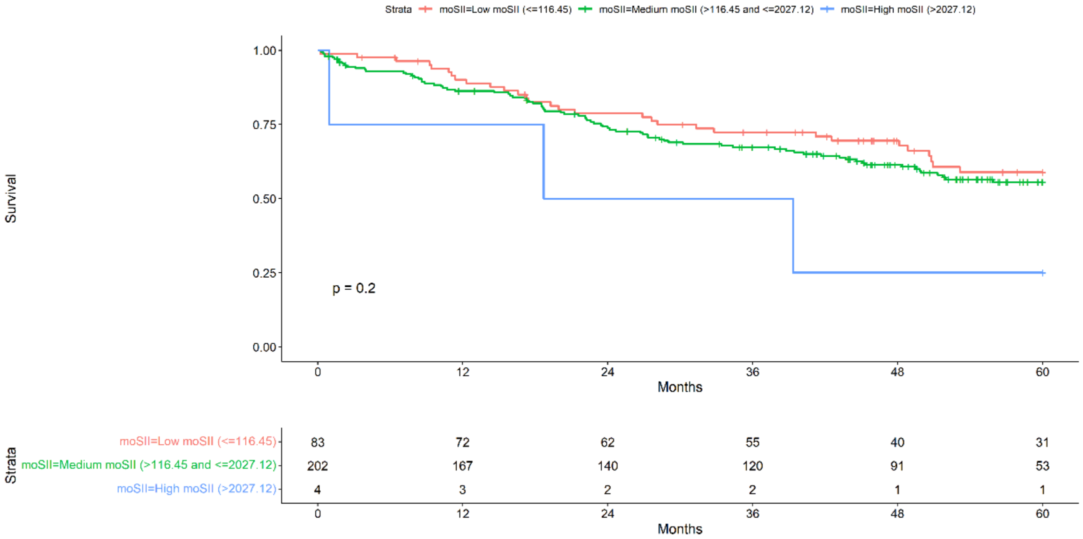

Stage III

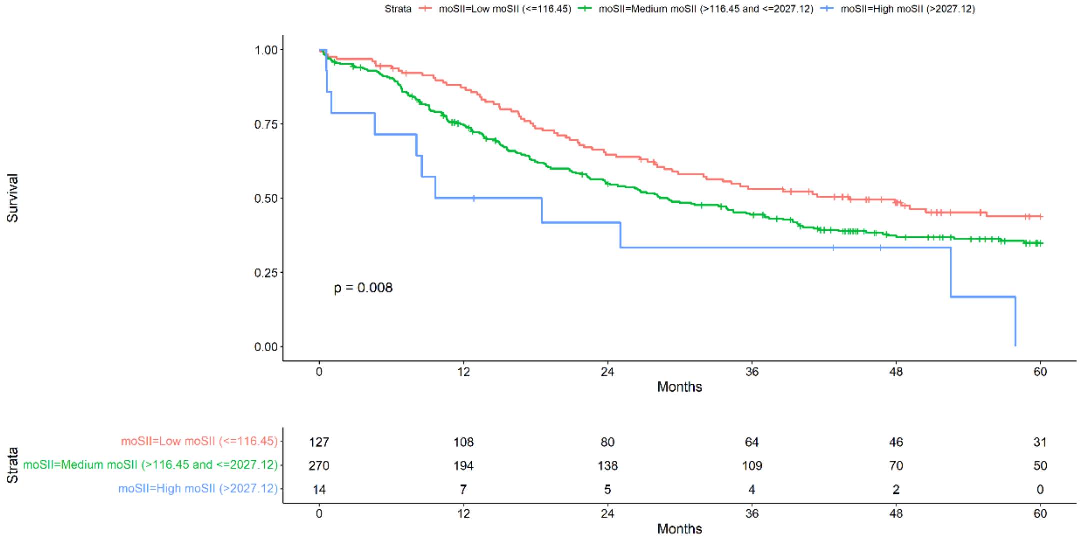

c) Overall survival for PNI

Stage I

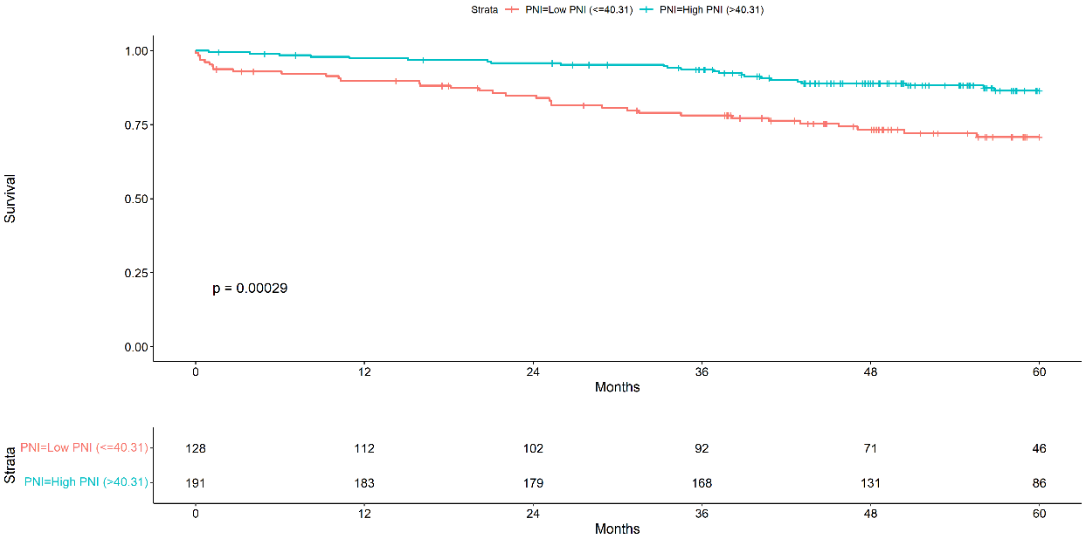

Stage II

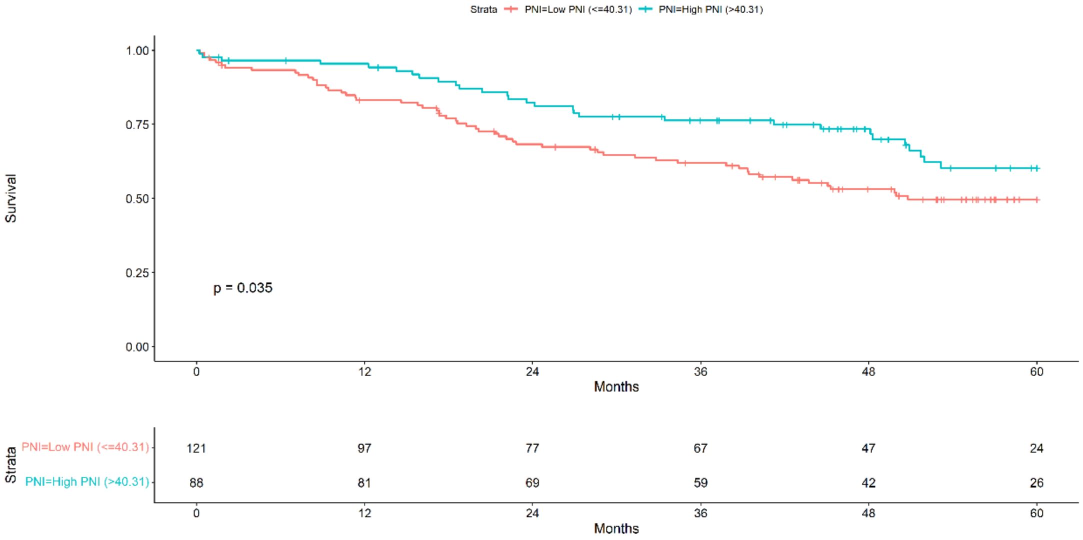

Stage III

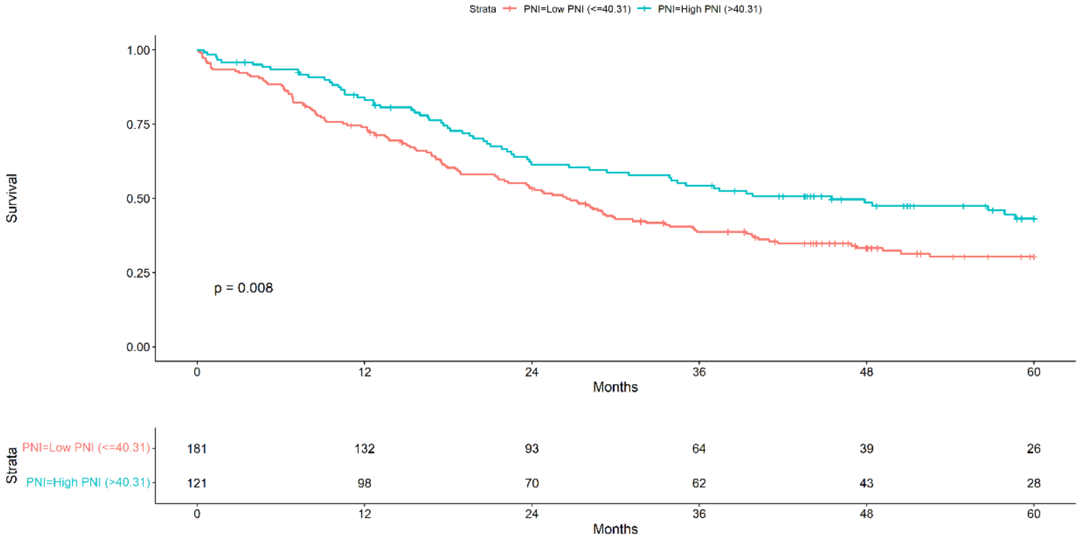

d) Disease free survival for NLR

Stage I

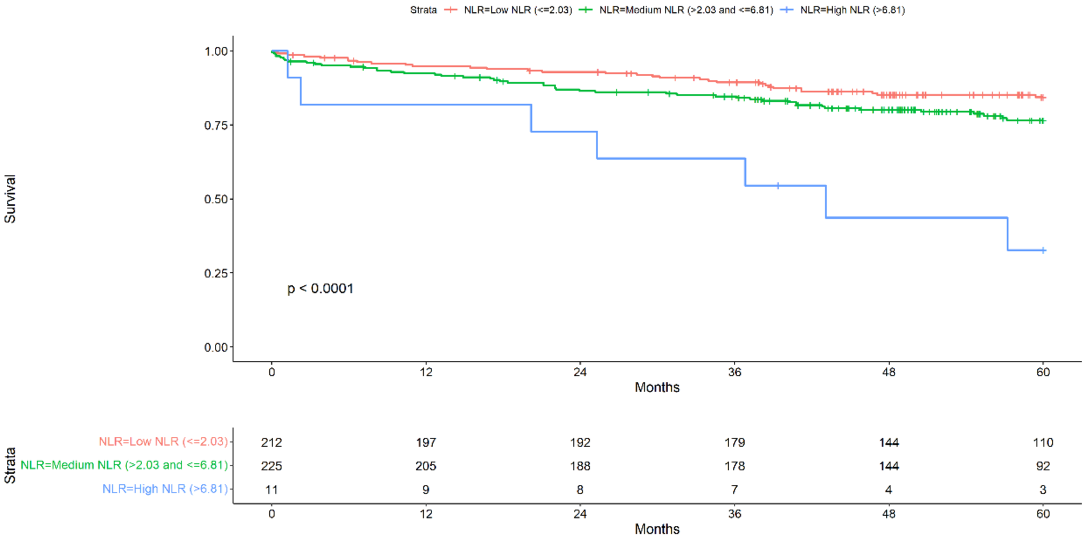

Stage II

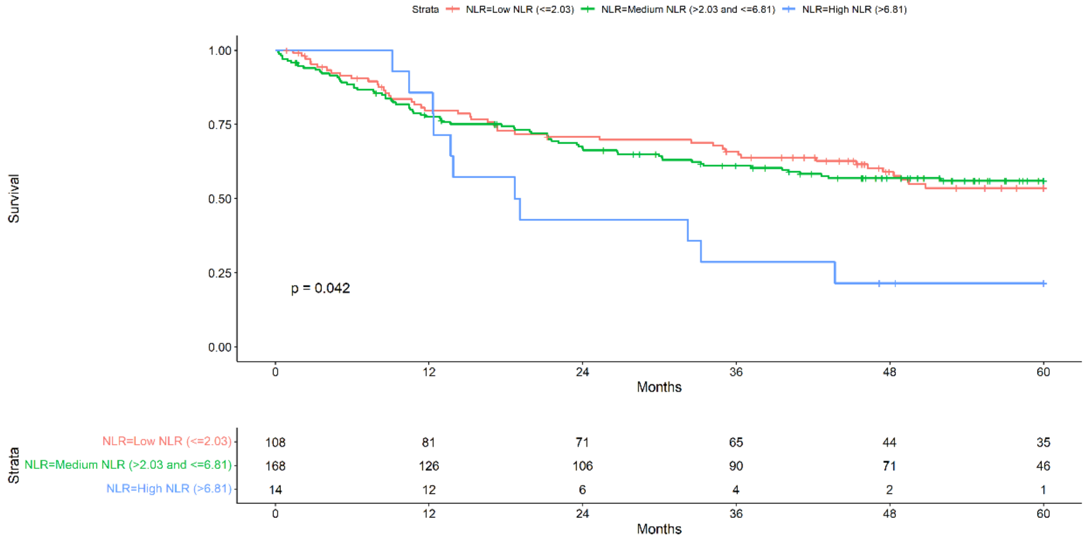

Stage III

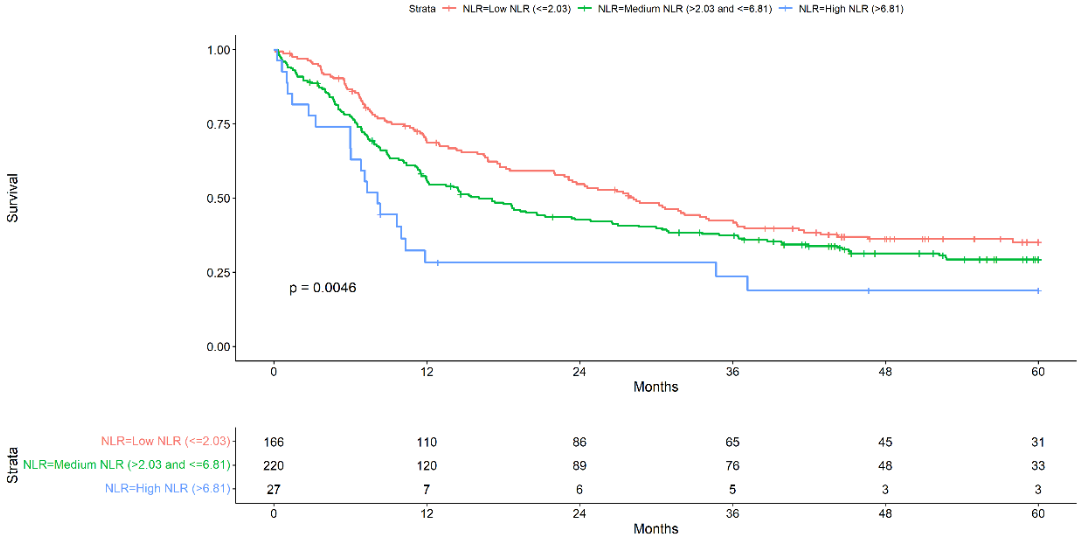

e) Disease free survival for moSII

Stage I

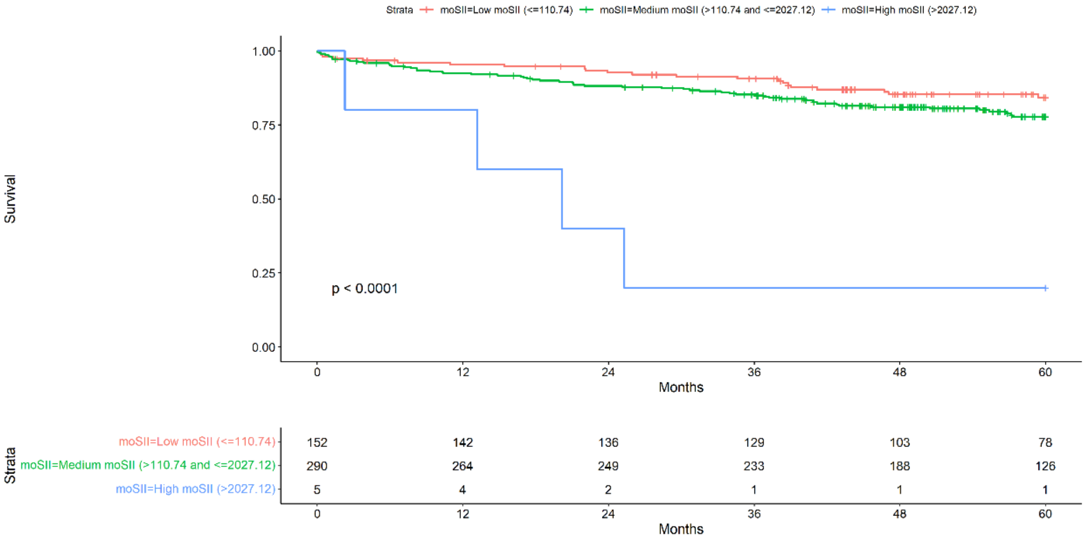

Stage II

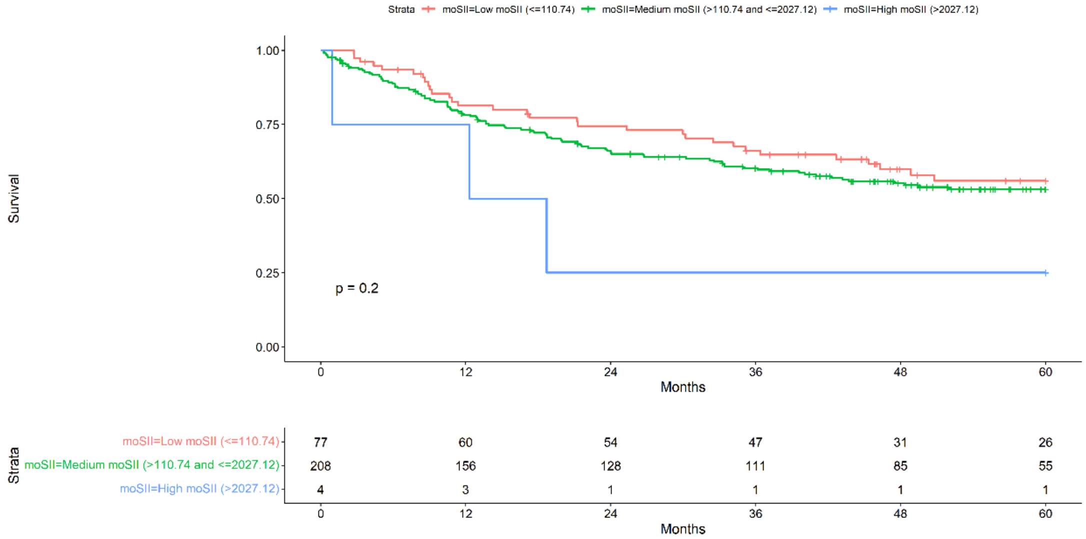

Stage III

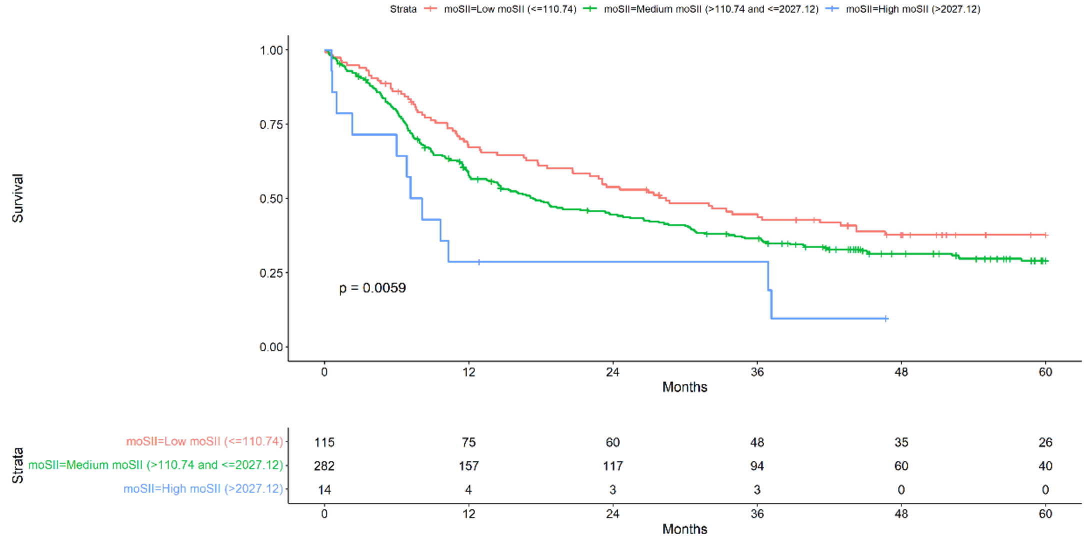

f) Disease free survival for PNI

Stage I

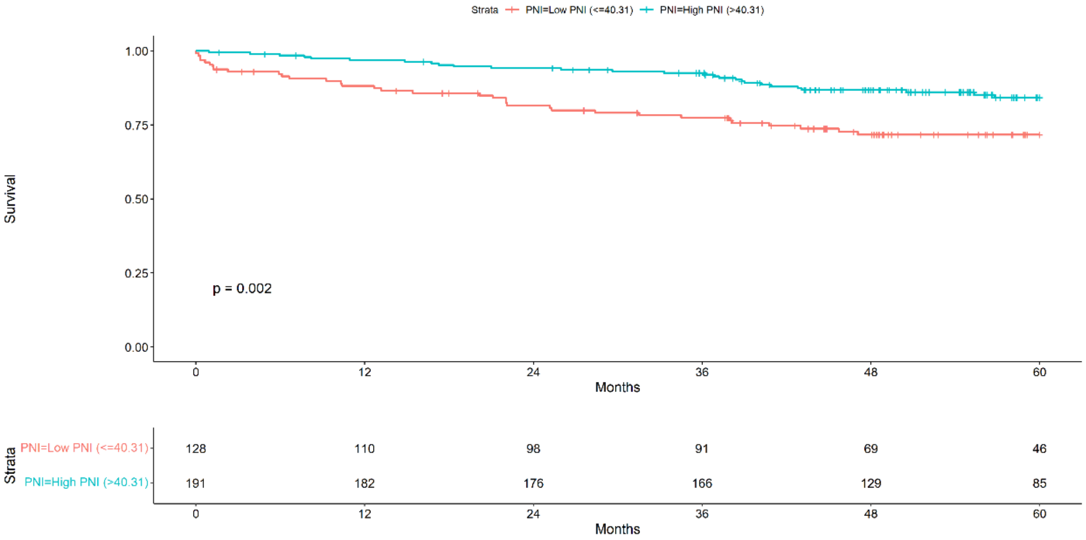

Stage II

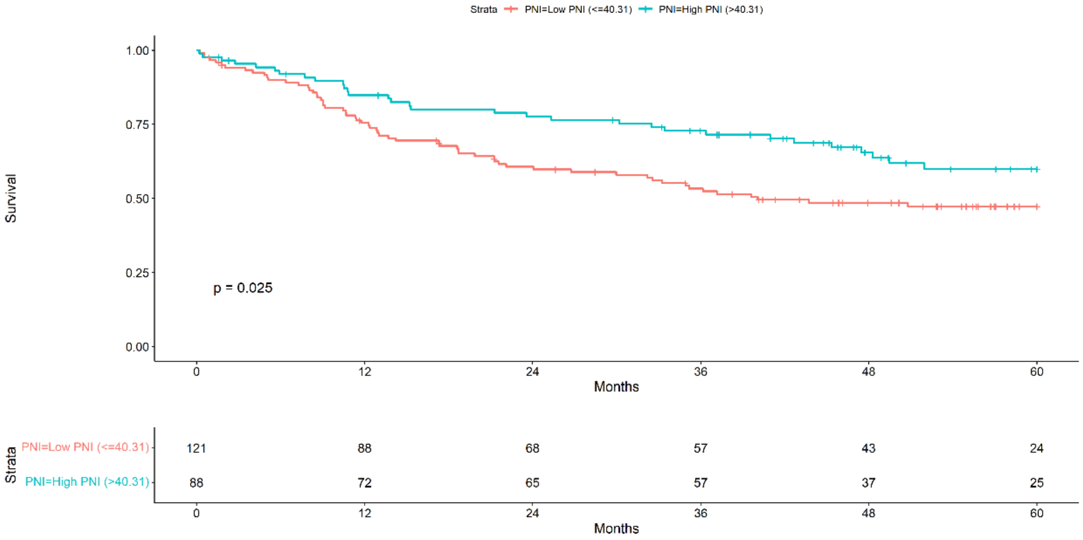

Stage III

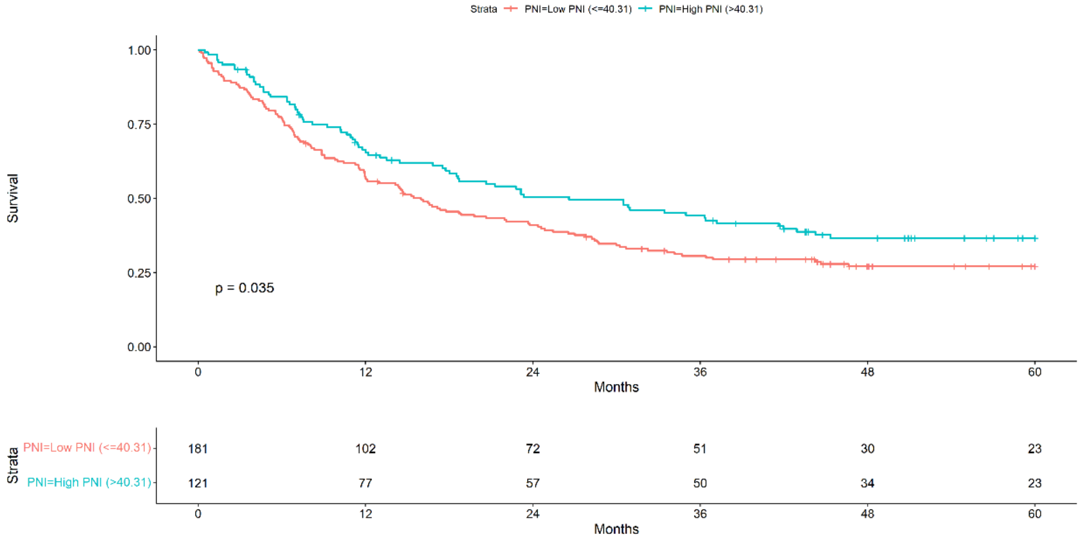

Supplementary Table S5: Multivariant Cox regression models to analyze the association of NLR, moSII and PNI on overall survival.

	**Model with NLR and moSII**			**Model with NLR and PNI**			**Model with moSII and PNI**			**Model with NLR, moSII and PNI**		
	**HR**	**95% CI**	**p-value**	**HR**	**95% CI**	**p-value**	**HR**	**95% CI**	**p-value**	**HR**	**95% CI**	**p-value**
**NLR**												
Low NLR (<=1.99)	Ref			Ref						Ref		
Medium NLR (>1.99 and <=5.91)	1.00	0.76, 1.30	>0.9	0.95	0.74, 1.22	0.7				0.81	0.59, 1.12	0.2
High NLR (>5.91)	1.37	0.89, 2.12	0.2	1.55	1.03, 2.34	0.034				1.21	0.73, 1.99	0.5
**moSII**												
Low moSII (<=116.45)	Ref						Ref			Ref		
Medium moSII (>116.45 and <=2027.12)	1.17	0.89, 1.55	0.3				1.12	0.87, 1.46	0.4	1.26	0.90, 1.78	0.2
High moSII (>2027.12)	1.74	0.93, 3.27	0.085				2.26	1.19, 4.30	0.013	2.04	0.96, 4.33	0.065
**PNI**												
Low PNI (<=40.31)				Ref			Ref			Ref		
High PNI (>40.31)				0.77	0.60, 0.99	0.045	0.78	0.60, 1.00	0.054	0.79	0.61, 1.02	0.070

HR = hazard ratio, CI = confidence interval, NLR = neutrophil-to-lymphocyte ratio, moSII = monocyte-systemic inflammation index; PNI = prognostic nutritional index.
All models adjusted by age, ECOG, tumour localization, Charlson index, CEA, body mass index, tumour stage and neoadjuvant therapy
Multivariant Cox regression models to analyse the association of NLR, moSII and PNI on overall survival. Model with NLR and moSII: Cox regression model using overall survival as dependent variable and NLR and moSII as independent factors adjusted by age, ECOG, tumour localization, Charlson index, CEA, body mass index, tumour stage and neoadjuvant therapy. Model with NLR and PNI: Cox regression model using overall survival as dependent variable and NLR and PNI as independent factors adjusted by the same factors. Model with moSII and PNI: Cox regression model using overall survival as dependent variable and moSII and PNI as independent factors adjusted by the same factors. Model with NLR, moSII and PNI: Cox regression model using overall survival as dependent variable and NLR, moSII and PNI as independent factors adjusted by the same factors.
